# From Probabilistic to Fuzzy Matching Record Linkage: A promising Transition

**DOI:** 10.23889/ijpds.v9i5.2624

**Published:** 2024-09-18

**Authors:** Mahmoud Azimaee, Gangamma Kalappa, Charlotte Ma, Nan Wang, Winnie Shen

**Affiliations:** 1 ICES; 2 University of Toronto

## Objective

 Probabilistic Record Linkage (PRL) heavily relies on manual intervention for gray area resolutions. This makes PRL extremely time and resource intensive. No matter how scientifically sound and robust PRL method is, it didn’t meet close-to-real-time data availability requirements at ICES.

## Approach

During a thorough evaluation and comparison process of three software and two methods of Record Linkage, ICES initiated a semi-design of experiment to select an optimal record linkage approach. For this experiment, a large Ontario data on 12 million individuals with required linkage variables plus valid Ontario health card numbers was selected. While the availability of the health card number enabled assessment of the accuracy of different approaches, the analysts were blinded to the correct health card numbers during the process. If manual intervention was required, it was repeated by two analysts to allow capturing human error.

## Results

PRL-based software needed the most personnel time to complete the process. Human errors were identified during the manual intervention due to subjective decisions by the analysts. Fuzzy Matching approach eliminated manual intervention but achieved comparable linkage rate to PRL while maintaining the same accuracy. The Fuzzy Matching software costs were higher; however, the data timeliness was significantly improved, and the clerical review costs and human error were eliminated.

## Conclusions

The Modernization of Record Linkage (MORL) project was a successful demonstration of the advantages of Fuzzy Matching over PRL method. However, the implementation of new approach at the organization level was a challenging change management.

